# Synthesis and Enhanced Phosphate Recovery Property of Porous Calcium Silicate Hydrate Using Polyethyleneglycol as Pore-Generation Agent

**DOI:** 10.3390/ma6072846

**Published:** 2013-07-15

**Authors:** Wei Guan, Fangying Ji, Qingkong Chen, Peng Yan, Ling Pei

**Affiliations:** Key Laboratory of Three Gorges Reservoir Region’s Eco-Environment, Chongqing University, Chongqing 400045, China; E-Mails: guanwei951030@126.com (W.G.); chenqingkong@126.com (Q.C.); yanpengstu@163.com (P.Y.); huashengtangjiang@126.com (L.P.)

**Keywords:** calcium silicate hydrate, porosity, hydroxyapatite, hydrothermal synthesis, solubility

## Abstract

The primary objective of this paper was to synthesize a porous calcium silicate hydrate (CSH) with enhanced phosphate recovery property using polyethyleneglycol (PEG) as pore-generation agent. The formation mechanism of porous CSH was proposed. PEG molecules were inserted into the void region of oxygen–silicon tetrahedron chains and the layers of CSH. A steric hindrance layer was generated to prevent the aggregation of solid particles. A porous structure was formed due to the residual space caused by the removal of PEG through incineration. This porous CSH exhibited highly enhanced solubility of Ca^2+^ and OH^−^ due to the decreased particle size, declined crystalline, and increased specific surface area (S_BET_) and pore volume. Supersaturation was increased in the wastewater with the enhanced solubility, which was beneficial to the formation of hydroxyapatite (HAP) crystallization. Thus, phosphate can be recovered from wastewater by producing HAP using porous CSH as crystal seed. In addition, the regenerated phosphate-containing products (HAP) can be reused to achieve sustainable utilization of phosphate. The present research could provide an effective approach for the synthesis of porous CSH and the enhancement of phosphate recovery properties for environmental applications.

## 1. Introduction

Phosphorus is one of the most important elements in the biosphere and a component of biological cells. This element is essential for storage of energy and composition of genetic materials [1,2]. The spontaneous migration and transformation of phosphorus is an irreversible cycle [3,4]. An essential element for organisms such as plants, phosphorous in the form of phosphate is a vital ingredient in fertilizers. Large-scale use of phosphate has depleted many of the natural resources of phosphorous. It is noteworthy that this precious and depleting resource will be exhausted as a result of increased consumption in the near future [5]. The problem of sustainable utilization of phosphorus has become a major global challenge [6,7]. Phosphorus in the wastewater mainly exists in the form of phosphate [8]. Thus, it is exigent and necessary to recover phosphate from wastewater. HAP crystallization processes have been considered as effective methods to recover phosphate from wastewater and could provide potential routes to solve this issue [9,10,11,12].

Calcium silicate hydrate (CSH), as crystal seed, has become a hot topic of intensive interest due to its sustained release of Ca^2+^ and OH^−^ in phosphate recovery from wastewater by producing hydroxyapatite (HAP) crystallites [13,14,15,16,17]. When Ca^2+^, OH^−^ and phosphate ions in the wastewater reach the supersaturation state, HAP crystallization can be produced spontaneously on the surface of CSH [18,19,20]. Thus, increasing the concentration of Ca^2+^ and OH^−^ in the wastewater is beneficial to enhance the HAP crystallization. However, the solubility of Ca^2+^ and OH^−^ of present CSH (including tobermorite and xonotlite and so on) is too poor to maintain the supersaturation state continuously due to their dense pore structure [13,21,22,23]. This is because the original dense pore structure can be blocked by HAP crystallization quickly at the beginning of reaction stage. The dense pore structure and poor solubility of CSH are therefore the two key factors of phosphate recovery [24]. 

In order to enhance the phosphate recovery property of CSH, it is significant to develop a pore-generation agent to produce porous CSH structure. Polyethylene glycol (PEG) is a kind of water-miscible polymer with low molecular weight [25,26]. This polymer has good dispersion and stability on the particle surface due to the adjustable hydrophilic character of PEG which is highly branched [27,28,29]. Furthermore, this polymer can be removed completely by incineration [30,31,32]. If the specific surface area (S_BET_) and pore volume can be increased using PEG as a pore-generation agent, the phosphate recovery property of CSH should be enhanced remarkably. Nevertheless, the detailed formation mechanism of porous CSH using PEG as a pore-generation agent has not been explored thus far. 

In the present study, in order to enhance the phosphorous recovery properties of CSH, porous CSH was synthesized using PEG as pore-generation agent. The detailed formation mechanism of porous CSH with PEG was examined. In addition, the structural features and the solubility of the new porous CSH were analyzed by a series of characterization tools. Finally, a new mechanism for the enhancement of phosphate recovery was proposed.

## 2. Experimental Section 

### 2.1. Materials

The CSH was made from industrial waste products (carbide residue). The CaO raw material (carbide residue, chemical constituents of carbide residue is shown in Table 1) and silicon raw material (silica, content of SiO_2_ >98%) were obtained from Chongqing Changshou Chemical Co. Ltd. PEG (the chemical formula is HO(CH_2_CH_2_O)*_n_*H, and molecular weight of this PEG is 2000) were obtained from Chengdu Kelong chemical Co. Ltd. The phosphate (P) solution was prepared by adding KH_2_PO_4_ (analytical reagent, Chongqing Boyi Chemical reagent Co. Ltd.) to solution with initial phosphate concentration of 100 mg/L. The said materials and chemicals were placed into sealed bottles for storage.

**Table 1:** Chemical components of carbide residue.

Chemical samples	Carbide residue							
Chemical Components	CaO	SiO_2_	Al_2_O_3_	SO_2_	MgO	Fe_2_O_3_	SrO	H_2_O
Contents/%	79.34	3.57	2.14	1.22	0.62	0.21	0.26	12.64

### 2.2. Preparation and Modification of CSH

#### 2.2.1. Preparation of CSH

(1) Carbide residue and silica were mixed, and the Ca/Si molar ratio was controlled at 1.6/1.0. The mixture was added to water to prepare 300 mL of slurry; (2) The slurry was hydrothermally reacted at 170 °C for 6 h, and the synthesis reaction was conducted at a stirring rate of 90 r/min; (3) Active slurry was taken out when the temperature was reduced to room temperature; (4) The hydrothermal reaction was carried out with a liquid/solid ratio of 30/1. The resultant products were dried at 105 °C for 2 h and then ground and sieved through a 200 μm mesh. This sample was labeled as CSH (PEG-0). The chemical reactions for the CSH sample production can be described as follows:

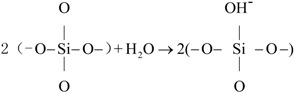
(1)

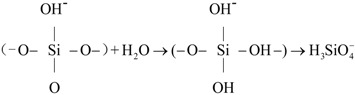
(2)

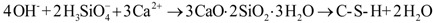
(3)

#### 2.2.2. Modification Using PEG

(1) The first three steps were the same as the first three steps of Section 2.2.1 to generate in active slurry; (2) After removal of slurry, PEG was added to five slurries (a weight percent relative to initial gel solution was 0.1%, 0.2%, 0.4%, 0.6% and 0.8%, respectively). These slurries were stirred for 1 h at 80 °C in a water bath with a stirring rate of 60 r/min; (3) Slurries were filtered and dried at 105 °C. The resulted sediments were put into the muffle furnace and heated at 500 °C for 2 h. The modified CSH could be obtained when the reaction was completed. These synthesized samples were labeled as CSH (PEG-0.1%), CSH (PEG-0.2%), CSH (PEG-0.4%), CSH (PEG-0.6%) and CSH (PEG-0.8%), respectively.

### 2.3. Solubility Experiments

The solubility was investigated by release of Ca^2+^ and OH^−^ in a series of batch experiments. For each experiment, 1 g of a sample was poured into 1 L of deionized water in a glass bottle, thus leading to a sample to solution ratio of 1 g/L. The bottle was placed on an agitation table and shaken at 40 r/min at a given temperature (20 °C). Samples of solution were taken out after 5, 10, 15, 20, 40 and 60 min of agitation. Ca^2+^ concentrations in solution were determined by EDTA coordination titration method (The relative derivation of data is 0.05%). pH values were measured by Precise pH papers (pH 7.0–10.0, San-ai-si reagent Co. Ltd., Shanghai, China. The accuracy of pH measurement is 0.1). 

### 2.4. Experiments on Phosphate Recovery from Synthetic Solutions

Phosphate recovery property of the as-synthesized samples and mechanisms of phosphate recovery were investigated in a series of batch experiments. The pH values of phosphate-content solution were in the range of 7.0–7.5 before the CSH sample was added into this solution. For each one, one glass bottle containing 1 L of a synthetic solution with initial phosphate concentration (100 mg/L) was prepared. Subsequently, 1 g of synthesized sample was put into this bottle, thus leading to a sample to solution ratio of 1 g/L. The bottle was placed on an agitation table and shaken at 40 r/min under given temperature conditions (20 °C) for 60 min. The solid samples after reaction were then separated from the removed synthetic solution, and were added again to synthetic solution with an initial phosphate concentration of 100 mg/L. This experiment was repeated six times until the phosphate concentration was kept unchanged with the addition of samples [17]. The residual phosphate concentration of the solution was measured according to the molybdenum blue ascorbic acid method (the relative derivation of data is 0.3%) with a Unico spectrophotometer (UV-2012PCS, Shanghai Unico Instruments Co., Ltd., Shanghai, China). The solid samples after reaction were weighed when separated from the removed synthetic solution and dried at 105 °C. Phosphate contents of the samples after phosphate recovery (*P*) were calculated by Equation (4), where *C_t_* is the remaining phosphate concentration in synthetic solution (mg/L), *v* is the volume of the solution (L), *w* is the mass of resultant sediment after phosphate recovery (mg) and *C*_0_ is the initial phosphate concentration (mg/L) [8,33].


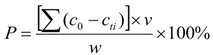
(4)

### 2.5. Characterization Instruments

The crystal phases of the sample were analyzed by X-ray diffraction using Cu Kα radiation (XRD, model XD-2 instrument, Persee, China). Field-emission scanning electron microscopy (FESEM, Japan) was used to characterize the morphology of the obtained products. Nitrogen adsorption–desorption isotherms were obtained on a nitrogen adsorption apparatus (ASAP-2010, USA). All the samples were degassed at 200 °C prior to measurements. Fourier transformed infrared spectroscopy (FT-IR, IR Prestige-21FT-infrared spectrometer, Shimadzu, Japan) in the transmission mode in the KBr pellet technique measured the resolution at 4 cm^−1^, scan 40 times. Particle Size Distributions were performed with BT-9300HT laser particle analyzer (measurement range: 0.1~1000 μm).

## 3. Results and Discussion

### 3.1. Specific Surface Areas (S_BET_) and Pore Structure

The S_BET_ and pore structure of the synthesized samples were investigated by adsorption–desorption measurements. As shown in Table 2, the S_BET_ of CSH (PEG-0.6%) increased to 352.96 m^2^/g compared to CSH (PEG-0) (86.92 m^2^/g). In comparison to CSH (0.28 cm^3^/g) the pore volume of CSH (PEG-0.6%) increased to 0.87 cm^3^/g. The CSH (PEG-0.6%) possessed the maximum S_BET_ and pore volume. However, the S_BET_ and pore volume of the modified CSH decreased when the concentration of PEG was either too low or too high. 

**Table 2:** Specific BET surface areas and pore parameters of synthesized calcium silicate hydrate (CSH) samples.

Samples	Total volume (cm^3^/g)	Peak pore diameter (nm)	S_BET_ (m^2^/g)
CSH (PEG-0)	0.28	12.97	85.92
CSH (PEG-0.1%)	0.20	10.28	77.95
CSH (PEG-0.2%)	0.28	9.34	118.77
CSH (PEG-0.4%)	0.40	10.79	148.11
CSH (PEG-0.6%)	0.87	9.83	352.96
CSH (PEG-0.8%)	0.31	10.17	119.94

**Figure 1:**
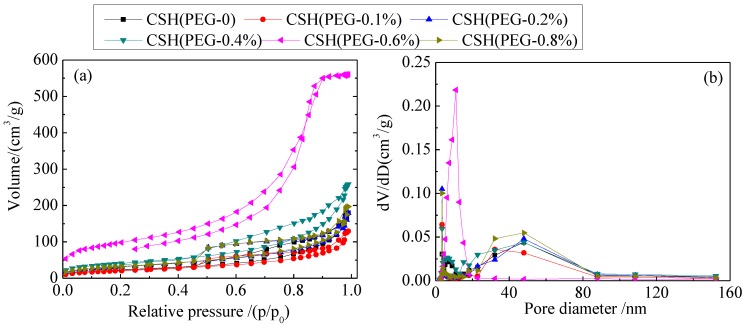
N_2_ adsorption–desorption isotherms (**a**) and pore-size distribution curves (**b**) of the synthesized CSH samples.

Figure 1a shows the N_2_ adsorption–desorption isotherms of the CSH samples. According to the Brunauer–Deming–Deming–Teller (BDDT) classification, the majority of physisorption isotherms can be grouped into six types. The isotherms of all the samples belonged to type IV, including the pore-size distributions in the mesoporous regions [34]. The shapes of hysteresis loops were of the type H3, which was associated with mesopores formed due to aggregation of plate-like particles [35].

Figure 1b shows the corresponding pore-size distribution (PSD) of the samples. For CSH (PEG-0), the PSD curve is bimodal with smaller (~3.51 nm) and larger (~47.92 nm) mesopores. For CSH (PEG-0.6%). The PSD curve exhibited small (~10.97 nm) mesopores. 

### 3.2. Formation Mechanism of the Porous Structure

**Figure 2:**
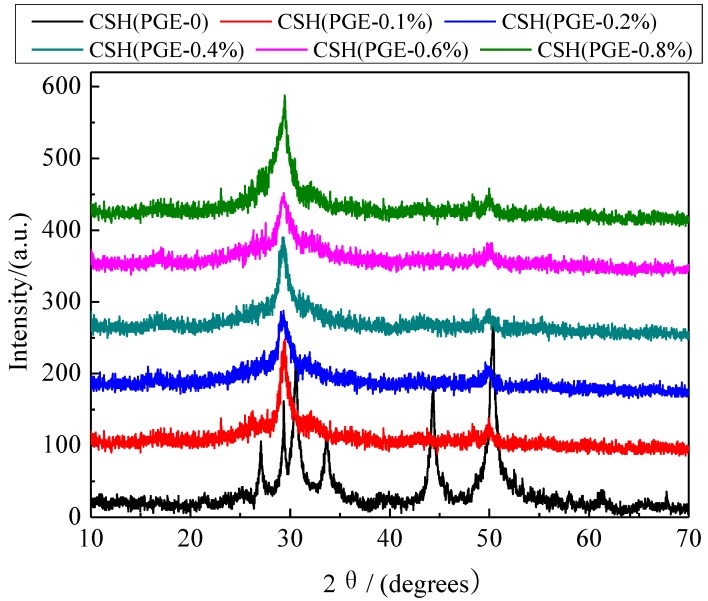
X-ray diffraction (XRD) patterns of CSH samples before and after modified by PEG.

The X-ray diffraction (XRD) patterns of CSH samples synthesized in the presence of different concentrations of PEG were compared (Figure 2). In the absence of PEG, the major phase of CSH (PEG-0) was Jennite (PDF card 18–1206, chemical formula Ca_9_Si_6_O_18_(OH)_6_·8H_2_O). The diffraction reflections were located at 8.40°, 29.92°, 31.78°, 35.74° and 49.70°, respectively. After modification, the characteristic reflections of CSH at 31.78° and 35.74° disappeared, and the intensity of the dominant reflection at 29.92° became weaker. This phenomenon indicated that the crystal structure of CSH was distorted and the crystallinity was decreased due to the incorporation of PEG. The characteristic reflection at 8.40° (002) disappeared after modification. The crystallinity declined obviously due to the fact that the PEG was inserted into the interlamination of CSH to increase the interlayer interval. According to the fitting of MDI jade 5.0, the crystallinity of CSH (PEG-0), CSH (PEG-0.1%), CSH (PEG-0.2%), CSH (PEG-0.4%), CSH (PEG-0.6%), CSH (PEG-0.8%) was 35.62%, 28.28%, 22.45%, 22.37%, 14.53% and 17.80%, respectively. 

**Figure 3:**
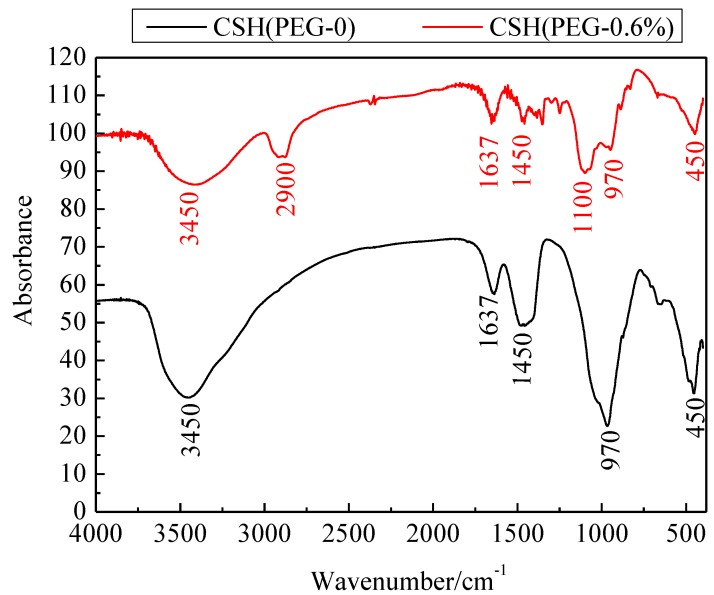
Fourier transform infrared spectroscopy (FI-IR) spectra of CSH (PEG-0) and CSH (PEG-0.6%).

In order to reveal the reaction mechanism between CSH and PEG, the CSH before and after inclusion of PEG (the concentration of PEG is 0.6%) was analyzed by Fourier transform infrared spectroscopy** (**FI-IR) spectra (Figure 3). Both the samples show a broad and sharp peak (O–H) at 3450 cm^−^^1^. The peak at 970 cm^−^^1^ could be attributed to the antisymmetric stretching vibration of Si–O–Si and the stretching vibration of O–Si–O. The absorption bands at 450 cm^−1^ could be assigned to the bending vibration of Si–O–Si. In addition, the bands at 1637 cm^−1^ in the spectra of these two samples belonged to the δ_HOH_ vibration of the coordinated water. The absorption band at 1450 cm^−1^ was assigned to the C–OH bending vibrations. These bands embody the unique silicon–oxygen tetrahedral structure of CSH [36,37,38]. However, the intensity of these bands became weaker when CSH was modified. This result suggested that the degree of order of CSH declined due to the modification of PEG. Except for these bands of CSH, the stretching vibration bands of C–H at 2900 cm^−1^ and stretching vibration bands of C–O at 1100 cm^−1^ were assigned to PEG [39]. This result indicated that PEG was a polymer containing a –CH_2_–CH_2_–O– group in its parent chain [40]. This polymer belonged to the non-ionic dispersant. Molecular structure of PEG included anchor groups and solvent chains [41]. 

**Figure 4:**
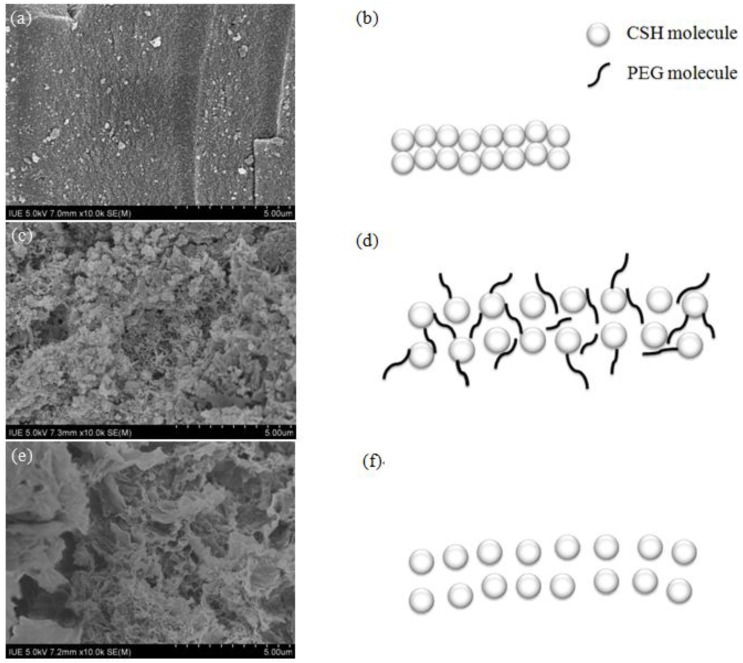
Field emission scanning electron microscopy (FESEM) photographs and schematic diagrams of CSH (PEG-0) and CSH (PEG-0.6%) during the modification process. (**a**) CSH (PEG-0), and the pore structure of this sample was dense before modification; (**b**) PEG molecules insert into the void region of oxygen–silicon tetrahedron chain or the layers of CSH (**c**,**d**); (**e**) CSH (PEG-0.6%), and porous structure was formed due to the residual space caused by the removal of PEG through incineration (**f**).

The modification process of CSH using PEG (the concentration was 0.6%) was suggested by FESEM images and schematic diagrams. The pore structure of CSH was dense before modification (Figure 4a,b). When PEG was added to the CSH slurry, PEG molecules were inserted into the void region of oxygen–silicon tetrahedron chain or the layers of CSH. Anchoring groups could be adsorbed on the surface of CSH particles, while the solvent chain was extended in the medium completely. Under this condition, the steric hindrance layer was formed to prevent the aggregation of solid particles (Figure 4c,d). After the modification process, porous structure was formed due to the residual space caused by the removal of PEG through incineration (Figure 4e,f).

**Figure 5:**
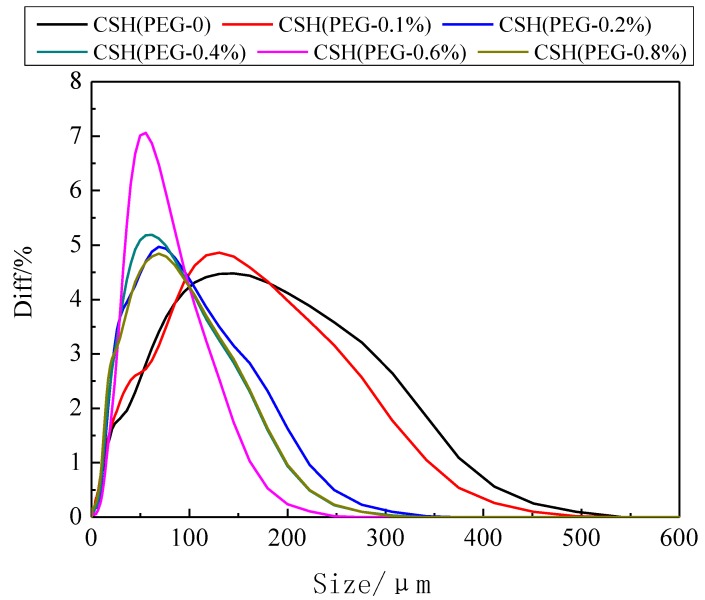
Particle size distributions of as-prepared CSH samples.

The pore volume and S_BET_ were not increased when the concentration of PEG was too either high or low. This phenomenon could be explained by analysis of particle size distributions. The particle size distributions of CSH before and after modification using PEG are shown in Figure 5. The particle size of CSH decreased due to the inclusion of PEG. The volume-based average diameter of CSH (PEG-0) and CSH (PEG-0.6%) were 111.41 μm and 56.24 μm, respectively. However, when the concentration of PEG was either too high or too low, the particle size of modified CSH was decreased because PEG is a non-ionic dispersant [42]. This dispersant changed the electrical properties of the powder surface and increased the electrostatic repulsion. The major influence of the concentration of PEG on the microstructure of CSH was the increased void space due to increasing the adsorption layer thickness. If the concentration of PEG was too low, the adsorption between the dispersant and particles became insufficient. At this condition, particles were aggregated by the interaction between the polymer chain end and the particles. On the contrary, if the concentration of PEG was too high, particles were also aggregated because the long chains were intertwined.

According to the above analysis, the main problem was the agglomeration of particles when CSH was modified by PEG. In the modification process, volume restriction effects and osmotic pressure effect could occur when the solid particles adsorbed enough PEG molecules. These two functions could produce the corresponding repulsive forces. These forces were large enough to offset the attractive potential caused by Van Der Waals forces. This repulsion could therefore prevent solid particles from approaching. In the modification process, not all the polymer chains were adsorbed on the interface. Partial absorption occurred with the rest of polymer chains being extended into the water to prevent the associating of Van Der Waals force among the CSH particles in space. Therefore, during the formation process of PEG–CSH sol, the water content within the CSH layer structure is likely to be reduced with the replacement of water molecules by hydrated PEG ions. Xu *et al.* reported that water is less strongly hydrogen bonded at low water concentration within the layered clay structure, and that these water molecules are clustered around exchangeable cations and are polarized by the close proximity to the exchangeable cation with the oxygen in the water molecule directed toward the metal cations [43]. Hence, the adsorption of PEG is likely to increase such polarization leading to a decrease in hydrogen bonding. Ha *et al.* further confirmed that Ca and Si in CSH are well intermixed at a sub-micron scale with PEG [44].

### 3.3. The Solubility and Dissolution Kinetics

**Figure 6:**
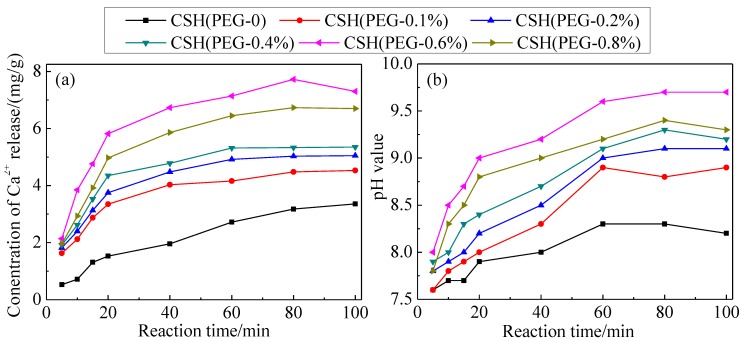
Variations of concentration of Ca^2+^ released from CSH samples (**a**) and pH values in deionized water (**b**).

Figure 6 shows the variations of concentration of Ca^2+^ released from the as-synthesized CSH samples and pH values in deionized water. Although the concentration of Ca^2+^ released from CSH (PEG-0.1%) was lower than for CSH (PEG-0), the solubility of modified CSH samples was increased with the increasing PEG concentrations. 

According to Figure 6a, CSH (PEG-0.6%) released more Ca^2+^ than the other CSH samples in the reaction mixture. This suggested that the dissolution of CSH (PEG-0.6%) was the primary reaction for the increase in the Ca^2+^ and pH of the solution. Compared with CSH (PEG-0), the concentration of Ca^2+^ released from CSH (PEG-0.6%) was increased from 3.36 to 7.30 mg/g. Figure 6b shows that the pH value of the solution could be kept at 9.7 by CSH (PEG-0.6%); however, other materials could only maintain the pH value at 8.2–9.3. As shown in Figure 6b, the final pH value of the aqueous solution apparently differs depending on the C–S–H adsorbent used. In such conditions, dominant phosphate species may differ, as well. At near neutral conditions (in the case of CSH (PEG-0)), H_2_PO_4_^−^ and HPO_4_^2−^ species may co-exist, and higher pH conditions (in the case of CSH (PEG-0.6%)), HPO_4_^2−^ might be the dominant species.

The experimental capacities of Ca^2+^ release are plotted according to the Avrami kinetic model equation [45], as shown in the following equation:

− ln(1 − *x*) = *kt^n^* (5)

where *k* is the kinetic constant, *n* is the characteristic constant of the solid, *t* is the reaction time (min) and *x* is calculated from the concentrations [*x* = *C_t_*/*C_max_*, *C_t_* is concentration of time *t* (mg/L) and *C_max_* is concentration at the maximum (mg/L)]. The characteristic constant *n* was 0.9067 through the fitting of −ln(1 − *x*) *vs. t^n^*. According to Equation (5), the value of the rate constant *k* could be obtained from a straight line with a slope. The obtained *k* values of CSH samples [CSH (PEG-0), CSH (PEG-0.1%), CSH (PEG-0.2%), CSH (PEG-0.4%), CSH (PEG-0.6%), and CSH (PEG-0.8%)] were 0.0455, 0.0425, 0.0522, 0.0647, 0.0827 and 0.0535, respectively. This result indicated that the trend changes in dissolution rate constant *k* and the S_BET_ showed a positive correlation. The trend between *k* and the particle size and crystallinity was negatively correlated. It can be inferred that the value of *k* was related to the S_BET_ (*S*) and particle size (*I*) and crystallinity (*D*). The correlation among these parameters could be established as follow:

*k* = *k*_0_ · *S^a^* · *I^b^* · *D^c^* (6)

where *k_0_*, *a*, *b* and *c* are constants. According to Figure 6a, *k_0_* = 0.3763, *a* = 0.4416, *b* = −0.5905, *c* = −0.7420. Then a relationship between *k* and *S*, *I*, *D* could be established:

*k* = 0.3763 · *S*^0.4416^ · *I*^(−0.5905)^ · *D*^(−0.7420)^ (7)

The relationship between *S*, *I*, *D* and the concentration of dissolved Ca^2+^ could be obtained by substituting Equation (7) into Equation (5):

− ln(1 − *x*) = 0.3763 · *S*^0.4416^ · *I*^(−0.5905)^ · *D*^(−0.7420)^*t*^0.9067^ (8)

According to Equation (8), the particle size and the crystallinity declined and the S_BET_ of CSH increased because of the inclusion of PEG. These changing trends enhanced the solubility of CSH.

### 3.4. The Phosphate Recovery Property

**Figure 7:**
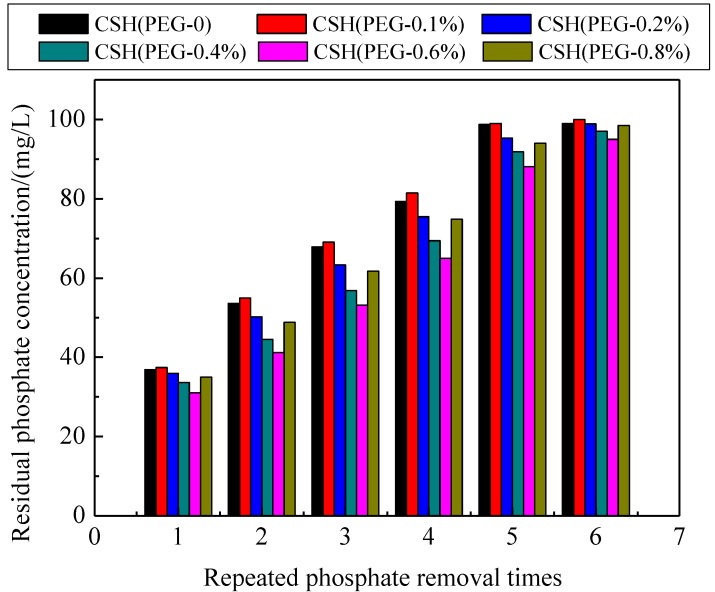
Changes of residual phosphate concentration by recycling phosphate removal of CSH samples.

**Figure 8:**
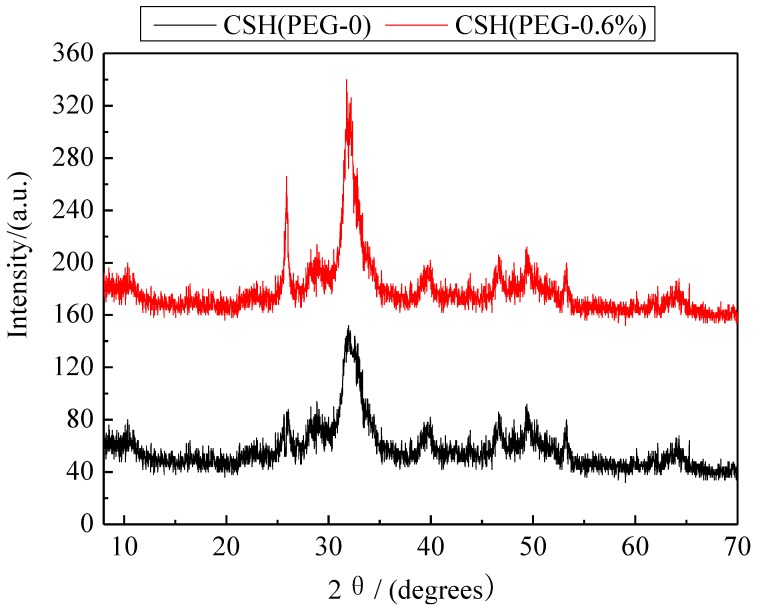
XRD patterns of regenerated product obtained by CSH (PEG-0.6%) and CSH (PEG-0).

The phosphate recovery properties of the CSH samples are shown in Figure 7. The quality and phosphate content of regenerated phosphate-containing products obtained by these samples are shown in Table 3. The phosphate recovery was enhanced by the use of PEG as pore-generation agent. The phosphate content of regenerated product obtained by CSH (PEG-0.6%) reached 15.40%, exceeding that of the regenerated product obtained by tobermorite (13%) and xonotlite (10%) [14,46]. This product could be reused as phosphate-containing product due to its high phosphate content (>15%) [47]. The XRD patterns of regenerated product obtained by CSH (PEG-0) and CSH (PEG-0.6%) are shown in Figure 8. After phosphate recovery, the sharp dominant reflection of HAP (PDF card 09–0432, chemical formula Ca_5_(PO_4_)_3_(OH)) were present at 25.88°, 31.76°, 49.50° and 53.26° instead of the dominant reflections of Jennite. This result indicated that HAP had been crystallized on the surface of CSH (PEG-0) and CSH (PEG-0.6%) during the phosphate recovery process. The value of the dominant reflections emerged at 25.88° and 31.76° of CSH (PEG-0.6%) was higher than that of CSH (PEG-0), thereby indicating that the HAP crystal produced better on the surface of CSH (PEG-0.6%). The major reaction of the HAP crystallization on CSH (PEG-0.6%) is as follows:

5*Ca*^2+^ + *OH*^−^ +3*HPO*_4_^2−^ → *Ca*_5_(*PO*_4_)_3_(OH)↓ + 3*H*^+^ (9)

According to Equation (9), CSH (PEG-0.6%), due to its capability of Ca^2+^ and OH^−^ release, can be used to recover phosphate from wastewater mainly by producing hydroxyapate (HAP) crystal. Besides, physical adsorption should be included in the phosphate recovery process due to the large S_BET_ of HAP.

**Table 3:** Quality and phosphate (P) content of regenerated products.

Samples	Quality (g)	Content of P (%)
CSH (PEG-0)	1.02	10.53
CSH (PEG-0.1%)	1.02	9.81
CSH (PEG-0.2%)	1.03	11.75
CSH (PEG-0.4%)	1.04	12.95
CSH (PEG-0.6%)	1.07	15.40
CSH (PEG-0.8%)	1.05	13.24

### 3.5. Mechanism of Phosphate Recovery Property Enhancement 

The supersaturation index (*SI*) of HAP can be expressed as follows:

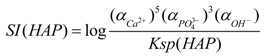
(10)
where *α*_*Ca*^2+^_, $\alpha_{PO_{4}^{\text{3−}}}$ and *α*_*OH*^−^_ are the ionic activity of *Ca*^2+^, $PO_{4}^{\text{3−}}$ and *OH*^−^, respectively. *K_sp_* (HAP) is the solubility product of HAP and this parameter is related to the temperature. The Gibbs function of precipitation reaction can be expressed as follows:

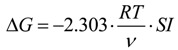
(11)
where G is the Gibbs free energy. *R*, *T* and *v* are a constant, absolute temperature and the number of ions, respectively. When *SI* = 0, Δ*G* = 0, the solution is in equilibrium state. When *SI* < 0, Δ*G* > 0, the solution is unsaturated. Under this condition, the precipitation reaction cannot occur. When *SI* > 0, Δ*G* < 0, the solution is in a supersaturation state. Under this condition, the precipitation can be formed spontaneously. Therefore, increasing the supersaturation is an effective way to enhance the phosphate recovery property when the reaction temperature is constant. Supersaturation in the phosphate solution was increased due to the enhanced solubility of the porous CSH. The porous structure of this CSH could provide favorable nucleation particles for the formation of HAP crystal nucleus. This crystal nucleus could enrich Ca^2+^, OH^−^ and phosphate ions to form HAP continuously. Therefore, the phosphate recovery property of CSH was enhanced due to the modification of PEG.

## 4. Conclusions 

Porous CSH was synthesized to enhance the recovery performance of phosphate using PEG as pore-generation agent. The formation mechanism of porous CSH was investigated. The large surface areas and pore volume, small particle size and low crystallinity in all contributed to the solubility enhancement. The enhanced solubility resulted in the increased supersaturation in phosphate solution. The increased supersaturation was beneficial to recover phosphate by producing HAP on the surface of CSH as crystal seed. The porous CSH exhibited highly enhanced phosphate recovery property, exceeding that of tobermorite and xonotlite. The phosphate content of regenerated products (>15%) is high enough to achieve the sustainable utilization of phosphate. The present work could provide an attractive and effective approach for enhancing the phosphate property of porous CSH.
